# The Benefits Outweigh the Risks of Treating Hypercholesterolemia: The Statin Dilemma

**DOI:** 10.7759/cureus.33648

**Published:** 2023-01-11

**Authors:** Azhar Hussain, Jasndeep Kaler, Sidhartha D Ray

**Affiliations:** 1 Pharmacology, Touro College of Pharmacy, New York, USA; 2 Medicine, Windsor University School of Medicine, Cayon, KNA; 3 Pharmaceutical and Biomedical Sciences, Touro College of Pharmacy, New York, USA

**Keywords:** statin, statin-induced myopathy, hdl-cholesterol, ldl-cholesterol, hmg-coa reductase, mitochondrial dysfunction

## Abstract

Cardiovascular diseases are one of the leading causes of death in the United States; therefore, primary and secondary prevention are of the utmost importance. In this regard, 3-hydroxy-3-methyl-glutaryl-coenzyme A reductase (HMG-CoA) reductase inhibitors, also known as statins, have been anointed as the primary treatment method for lowering cholesterol to prevent cardiovascular diseases. Statins decrease the low-density lipoprotein (LDL) cholesterol and triglycerides in the body, thus lowering the total body cholesterol levels. Despite the benefits associated with statins, it is essential to understand the adverse effects of these drugs. Myotoxicity and statin-associated muscle symptoms are the most common adverse effects of statins. The impairment of mitochondrial function is another adverse effect that can lead to hepatic dysfunction, neurocognitive effects, and potentially the new onset of diabetes. The exact pathophysiology of these side effects is still not fully understood. However, several mechanisms have been proposed, although there is significant overlap among the hypothetical propositions. Understanding the overall outcomes of each of these adverse effects can allow a healthcare practitioner to carefully map out whether statin administration should be used to prevent hypercholesterolemia in the body. The adverse effect of statins is dependent on both the dose and the type of statin used. Lipophilic statins tend to possess a more remarkable ability to infiltrate membranes; they have been hypothesized to cause statin-induced myopathies as well as neurocognitive effects by significantly crossing the blood-brain barrier. In summary, this review has focused on the mechanistic and clinical aspects of this statin class of medication. Proposed mechanisms for different adverse effects associated with statins remain a focus of this communication.

## Introduction and background

It is estimated that every 34 seconds, one individual in the United States will die from cardiovascular disease [1]. Cardiovascular diseases are a group of disorders involving the heart and blood vessels. Different cardiovascular diseases may present with diverse underlying pathologies, while risk factors and treatments may be very similar. Some leading risk factors for cardiovascular diseases are hypertension, high levels of low-density lipoprotein (LDL), diabetes, smoking, obesity, or a sedentary lifestyle [2]. High levels of LDL cholesterol can double an individual’s risk of heart disease [1-2].

Cholesterol is categorized based on the type of lipoprotein that carries it between cells. Among these, high-density lipoprotein (HDL) cholesterol, often referred to as the "good cholesterol" as at healthy levels, may bring protection against myocardial infarctions and strokes [2,3]. In contrast to HDL, LDL cholesterol is often referred to as "bad cholesterol." LDL cholesterol is the variation of cholesterol that contributes to fatty buildups in and around arteries, leading to the narrowing of the arteries. Atherosclerosis is the buildup of fats, cholesterol, and other substances in and around the artery walls and, thus, increases the risk of cardiovascular diseases [3]. HDL carries cholesterol from the arteries and tissues back to the liver, where it is metabolized and eliminated from the body [2,3]. Even though HDL carries cholesterol away from the arteries and out of circulation, it does not ultimately help decrease LDL cholesterol. HDL carries only one-third to one-fourth of the blood cholesterol in circulation [3]. Although nearly 86 million adults in the United States suffer from hypercholesterolemia and could benefit from taking appropriate medications to manage high LDL cholesterol, only about half are doing so [2]. The group of medications responsible for reducing LDL cholesterol levels acts by inhibiting the rate-limiting enzyme 3-hydroxy-3-methyl-glutaryl-coenzyme A reductase (HMG-CoA reductase) [4]. HMG-CoA reductase is a rate-limiting enzyme that catalyzes a reaction early in the mevalonate pathway. Inhibition of HMG-CoA reductase reduces cellular cholesterol synthesis and inhibits the production of other products that are generated through the same pathway. A few of these other products include coenzyme Q10, heme-A, and isoprenoids [5-7]. Isoprenoids play a role in post-translational modification products, which are accomplished via a process through which hydrophobic molecules are attached to proteins to activate them [7]. The attachment of hydrophobic molecules to proteins is done through the process of prenylation [5-7].

**Figure 1:**
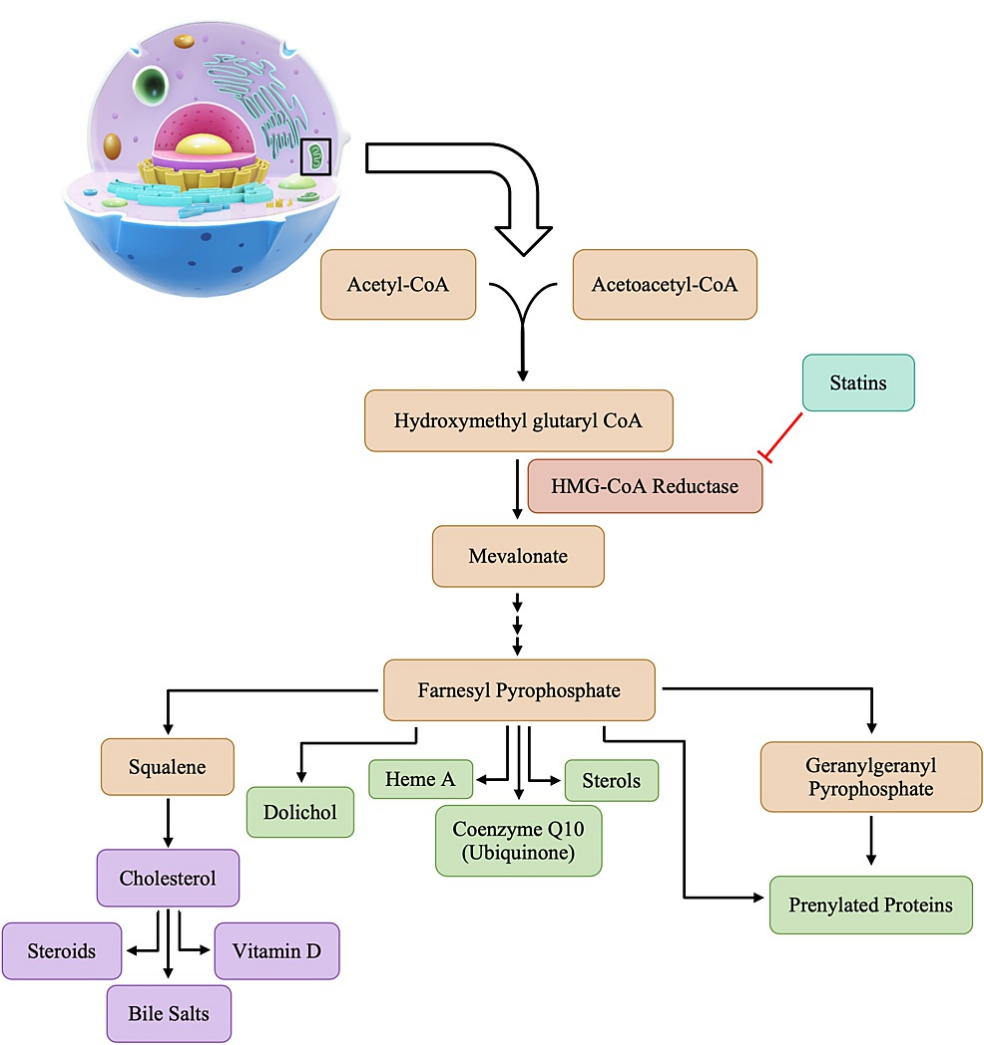
Products of cholesterol biosynthesis

The mevalonate pathway is an essential metabolic pathway for providing cells with bioactive molecules, pivotal for cellular processes [6]. The synthesis of cholesterol occurs in the cytoplasm and in the membrane of the endoplasmic reticulum of all human tissues, including the liver, intestine(s), adrenal cortex, and reproductive tissue [8]. Despite ongoing cholesterol synthesis occurring in human tissues, the liver produces about 70% of the total body cholesterol. Once the liver ceases to produce anymore, blood cholesterol levels will drop [8-9]. As shown in Figure 1, cholesterol biosynthesis begins with the condensation of acetyl-CoA with acetoacetyl-CoA, forming β-hydroxy-β-methylglutaryl coenzyme A (HMG-CoA) in a reaction that is catalyzed by the enzyme hydroxymethyl glutaryl coenzyme A (HMG-CoA) synthase [8]. The HMG-CoA reductase will then convert HMG-CoA to mevalonate, as shown in Figure 1. The conversion of HMG-CoA to mevalonate is the first rate-limiting step in the pathway of cholesterol synthesis [9]. After the production of mevalonate, a series of reactions catalyzed by various other enzymes occur, and the end products of these reactions include cholesterol, amongst many others. Cholesterol further facilitates the production of steroids, vitamin D, and bile salts, all of which are essential for normal bodily functions.

Statins competitively inhibit HMG-CoA reductase via a catalytic mechanism [6]. Statins will occupy the catalytic portion of the enzyme HMG-CoA reductase, which is normally specifically bound to the substrate HMG-CoA, thus blocking access of the substrate to the active site of HMG-CoA reductase [10]. While lowering cholesterol levels is the primary therapeutic goal of statin administration, decreased production of other downstream products is also noted [7]. By interrupting cholesterol synthesis in the liver, statins activate the production of microsomal HMG-CoA reductase and cell surface low-density lipoprotein (LDL) receptors, resulting in an increased clearance of LDL from the bloodstream, reflected in decreased blood LDL levels ranging anywhere from 20-55% [10]. Statins have revolutionized the treatment of patients with a high risk of cardiovascular diseases. The decision to use statins in patients with a history of cardiovascular disease is far more clear-cut than the choice of administering statins in high-risk individuals. Due to the pleiotropic nature of statin drugs, administration of a statin in individuals with a history of cardiovascular diseases may deter the development of any further conditions. However, the administration of statins for high-risk patients such as those with any other comorbidities, such as immunosuppression, autoimmune conditions, etc., must be much more carefully thought through. In patients with such conditions, the adverse profile of statins may cause a patient more harm than good. Due to the profile of adverse effects, determining whether statin therapy should be administered requires a harm versus benefit analysis on the part of healthcare practitioners. A study conducted by Pinal-Fernandez et al. concluded that lowering LDL cholesterol by 2 mmol/L for 5 years in 10,000 patients would reduce the rate of any major vascular events by 10% in secondary prevention and 5% in primary prevention [7]. Primary prevention is noted in individuals of lower risk, individuals with no history of cardiovascular events, or those who do not present with any major risk factors. The focus of secondary prevention is decreasing the risk of future cardiovascular events in individuals with multiple risk factors, a history of cardiovascular disease, or major vascular events. In addition to their known cardiovascular and anti-inflammatory effects, statins have been accredited with several other benefits [7,10]. There is also a risk of increased adverse effects in patients with multiple medical co-morbidities or in cases of chronic statin use [4]. Patients with medical co-morbidities who are treated with statins must do so cautiously, as the profile of adverse effects may be greater. Furthermore, the risk of drug-drug interactions may cause a greater and more detrimental effect on the body. Due to such possibilities, physicians must pay more attention to the worsening condition of patients upon statin administration.

**Figure 2:**
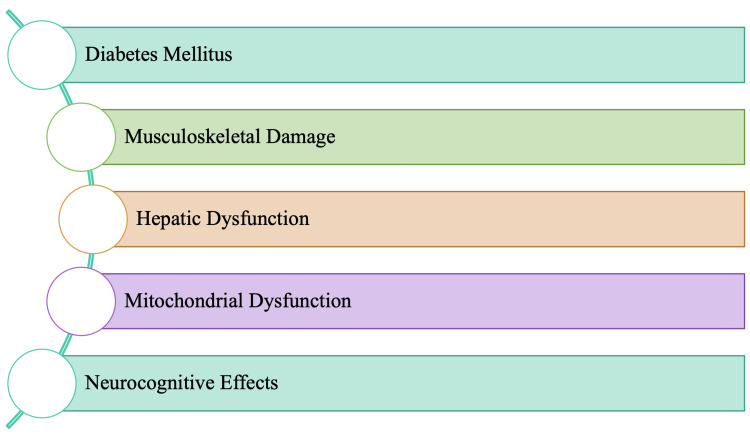
Adverse effects of statins

Although adverse effects with statin use are rare, the most common adverse effects that occur are noted in Figure 2. Due to some of these adverse effects, rates of adherence to statin therapy tend to be poor. Furthermore, adherence rates tend to be lower early on in the initiation of treatment, with nearly 75% of primary prevention patients stopping the drug within the first two years [11-12]. Statins' best-recognized and most reported adverse effects are of musculoskeletal origin, specifically myositis [5]. Many adverse effects associated with statin use tend to resolve upon discontinuation of the drugs. However, statin discontinuation does not fully resolve muscle effects [5,11]. The mitochondrial dysfunction noted in Figure 2 is associated with decreased levels of both coenzyme Q10 (CoQ10) and heme-A. Both CoQ10 and heme-A levels are essential for optimal mitochondrial function, specifically concerning the electron transport chain and oxidative phosphorylation.

Cholesterol is critical for vitamin D production, as well as other hormones and the production of bile. Statins block the production of bile acids, which are necessary for the absorption of fat and the maintenance of the microbiome in the gastrointestinal system. Vitamin D is critical for immune function, amongst many other essential physiological functions, such as protection against other metabolic syndromes. Blockade due to statins can cause the presence of gastrointestinal symptoms as well, which may be as mild as indigestion, constipation, or diarrhea. Blocking HMG-CoA reductase causes a decrease in the level of cholesterol in the brain, leading to adverse neurological adverse effects, as mentioned in Figure 2. In rare cases, long-term, chronic statin therapy patients have been noted to develop peripheral neuropathy. Another adverse effect is the onset of diabetes, which appears soon after starting statin therapy. It is crucial to note that the risk of new-onset diabetes is directly proportional to the dose of statin administered [7]. Interestingly, however, most diabetics have either hypercholesterolemia or are prone to it; therefore, statin use has become a standard recommendation. Hepatic dysfunction primarily occurs within the first three months of therapy and is also dose-dependent, which resolves with time in most cases [8]. Ongoing exploratory investigations may eventually provide a link between statin exposure and the new onset of diabetes.

While statins are highly effective in reducing the risk of major cardiovascular events by significantly downregulating cholesterol biosynthesis, the adverse effects can be detrimental in patients with pre-existing conditions. Different statins have different potencies, resulting in differing adverse effect profiles. Some statins, such as rosuvastatin, may be more potent than atorvastatin. However, both agents may be significantly more potent than simvastatin, lovastatin, pravastatin, and fluvastatin [8,13]. In such cases, statin therapy must be monitored closely.

This manuscript aims to summarize the probable pathophysiology of adverse effects for each statin, as noted in Figure 2. In doing so, the aim is to provide an understanding of the risks that may be associated with statins. Awareness and extensive knowledge of each adverse effect allow for appropriate treatment modification(s) for optimal outcomes to ensure improved quality of patient care and reduced patient morbidity.

## Review

While statins tend to be safe for use in most patients, there is an increased risk of adverse effects from long-term statin use in individuals with pre-existing conditions. The inhibition of HMG-CoA reductase blocks cholesterol biosynthesis, causing a halt in the production of other crucial molecules. While blocking cholesterol biosynthesis proves to be beneficial in reducing the risk of cardiovascular diseases, research suggests that it leads to the decreased production of hormones, along with vitamin D. Statins have been noted to produce a small but statistically significant decrease in the levels of testosterone and other sex hormones such as estrogen and progesterone [4,8]. Because cholesterol is a precursor of sex hormone biosynthesis, any synthesis blockade will consequently cause dysregulation of levels of sex hormones amongst individuals. A decrease in testosterone levels may be better acknowledged in males through decreased libido and erectile dysfunction, amongst other sexual dysfunctions. In this context, the overall cardiovascular risk of each patient should be assessed, which may allow clinicians to better assess and understand the considerable risk versus benefit analysis [4]. As such, it is imperative to understand the underlying pathophysiology of each adverse effect to ensure that the benefit of statin administration outweighs the risk for each patient.

**Figure 3:**
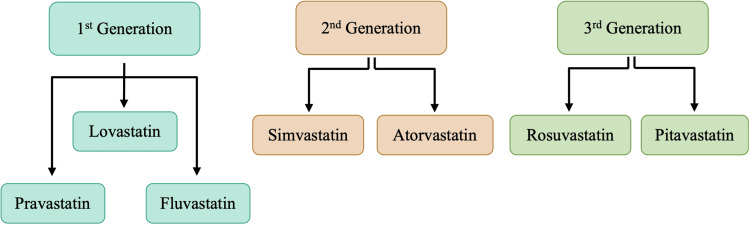
Classification of statins

Statins can be classified based on their increased potency and efficacy in lowering plasma LDL cholesterol concentration or their physicochemical properties. Different generations of statins have been denoted with differing potency and efficacy levels. Figure 3 is a visual representation of the classification of statins based on these two properties. The first generation of statins was introduced to the market during the late 1980s and 1990s [8,13]. This statin generation is pravastatin, lovastatin, and fluvastatin, and this class of statins has the lowest potency [8]. Among the first-generation statins, pravastatin was the most studied and proved to be effective in both primary and secondary prevention. Furthermore, fluvastatin and pravastatin received the most attention as first-generation statins, as they had the lowest drug-drug interaction risk [13]. Fluvastatin and pravastatin are not metabolized by the cytochrome p450 (CYP450) isoenzymes and, thus, are used as an alternative in patients who are intolerant to potent statins [8]. The second generation of statins consists of simvastatin and atorvastatin, and both are noted as the best-selling statins available on the market. Second-generation statins had higher efficacy than first-generation statins, as daily doses of only 10 mg atorvastatin and 20 mg simvastatin caused greater than a 30% lowering of LDL compared with the 20-40 mg daily doses of first-generation statins [13-14]. The third generation is noted as the "super statins," as they have the highest potency and efficacy. These third-generation statins are used as an alternative to the other statins on the market in high-risk patients who are more likely to develop statin intolerance [8]. 

Not mentioned in Figure 3 is another third-generation statin, cerivastatin, that has now been withdrawn from the market due to a greater incidence of fatal rhabdomyolysis than seen with other statins. Statins can also be classified based on their physicochemical properties, such as hydrophilic or lipophilic, depending on their ability to dissolve in water or a lipid-containing solution. Lipophilicity and hydrophilicity can ultimately determine the absorption and excretion of a drug. In this respect, absorption is faster with lipophilic drugs, whereas the ease of renal excretion is greater for hydrophilic statins [14]. The hydrophilic or lipophilic nature of statins can determine their entrance into cells and movement throughout the body. Most of the statins currently available on the market are lipophilic in nature, with the exceptions being rosuvastatin and pravastatin, the only hydrophilic statins.

The lipophilic statins can easily pass through and enter cell membranes, interacting with the surrounding acyl chains [4,14]. By contrast, hydrophilic statins remain associated with the polar surface of the cellular membrane. They usually require protein transporters to enter the cell to accomplish inhibition of HMG-CoA reductase [14]. Because lipophilic statins can travel deeper into the membranes, they are associated with more extrahepatic manifestations, such as a higher risk of statin-associated muscle symptoms. In contrast, it is probable that hydrophilic statins have less muscle penetration and therefore pose a lower risk. However, an observational cohort study concluded that hydrophilic statins were not better tolerated [14-15]. Due to the increased diffusibility of lipophilic statins, it has been hypothesized that they could induce a higher risk of neurocognitive effects, given their increased ability to cross the blood-brain barrier (BBB) [14]. Further studies and research must be conducted to confirm or reject this hypothesis. It can also be postulated that lipophilic statins are more readily involved in statin-induced adverse effects, and this can be largely attributed to their physicochemical properties.

Statins are administered orally and absorbed from the intestine, though they undergo extensive first-pass metabolism within the liver, reducing their systemic bioavailability to 5-50% [8]. Within systemic circulation, the statins will bind to albumin and also differ substantially with respect to half-life and volume of distribution [7-8]. The predominant route of metabolism of most statins is through the CYP450 system, with atorvastatin, lovastatin, and simvastatin metabolized through isoform CYP3A4 and fluvastatin metabolized through isoform CYP2C9 [4,13]. Pravastatin is metabolized largely through sulfation, while rosuvastatin is removed largely via biliary excretion [8,13-14]. Several other drugs are also metabolized via the CYP450 system; as such, it is essential to monitor the levels of other drugs to prevent drug-drug interactions. Some medications can also inhibit the CYP450 system, causing statin metabolites to increase in systemic circulation and, thus, leading to a more remarkable presentation of adverse effects. Adverse effects, such as myopathy, are noted more commonly in patients who receive higher doses of statins, which lead to higher plasma levels of active statin metabolites, especially in the first year of treatment or once the dosage has been increased [8,13].

Diabetes mellitus

Large randomized clinical trials have demonstrated an increased risk of developing diabetes mellitus in patients taking statins [7]. The attributable excess risk of developing diabetes has been estimated to be about 10-20 per 10,000 patients treated yearly, similar to the risk of developing a significant myopathy [7,16]. A meta-analysis conducted by Sattar et al. of 13 trials concluded a 9% increased risk of new-onset diabetes after a median four-year follow-up [16]. Trials and meta-analyses reported by Brault et al. suggest that the risk of new-onset diabetes is different amongst the different types of statins. Furthermore, a more recent meta-analysis reported that both the dose and the type of statin affect the risk of new-onset diabetes [17]. Evidence suggests that compared to pravastatin, patients treated with simvastatin presented a 10% increased risk of new-onset diabetes [17-18]. Rosuvastatin presents an 18% increased risk, and atorvastatin presents a 22% increased risk of new-onset diabetes [18]. In this regard, diabetics that are already on statins for cholesterol control could be of significant concern.

**Figure 4:**
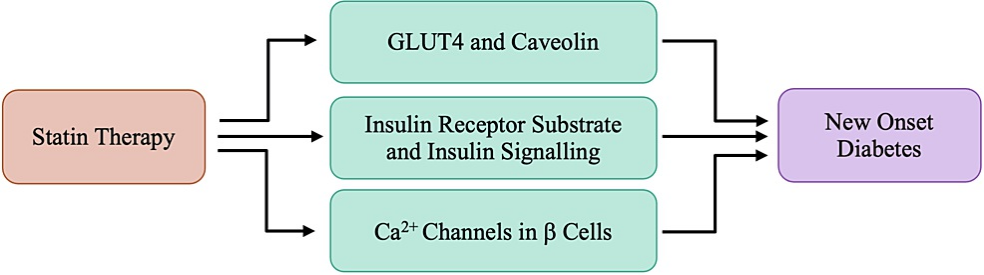
Proposed mechanisms of statins and new-onset diabetes

While the exact pathogenesis of statin-induced diabetes remains unknown, Figure 4 provides proposed mechanisms allowing insight into the precise association between statins and new-onset diabetes. Each proposed mechanism is associated with the effects of statins inhibiting HMG-CoA reductase.

Glucose uptake in adipocytes and striated muscle cells is mediated through a channel referred to as glucose transporter 4 (GLUT4), which is responsible for peripheral insulin-mediated glucose influx. Insulin-receptor tyrosine kinase phosphorylation is the signaling cascade's initiating event responsible for the recruitment of GLUT4 from its intracellular storage to the plasma membrane surface [17]. Nakata et al. reported a decrease in GLUT4 expression in 3T3L1 adipocytes after treatment with clinical doses of atorvastatin for 15 weeks [19]. 3T3L1 adipocytes are from a cell line of adipocytes specifically used to study adipogenesis. A decrease in caveolin-1 is also noted in the later stages of adipocyte differentiation. Caveolin-1 is a vital plasma membrane protein in caveolae-rich regions where GLUT4 is translocated upon stimulation by insulin [17,20]. Caveolae are specialized membrane microdomains within the plasma membrane of most cell types. However, they are most abundant in adipose tissue [20]. In recent studies, caveolin-1 and caveolae microdomains have emerged as potentially critical regulatory elements in the control of insulin signaling [17,20]. Takaguri et al. separated the cytosol and plasma membrane layers of 3T3L1 adipocytes to study the translocation of GLUT4 following the administration of atorvastatin. They concluded that following treatment with atorvastatin, the total concentration of GLUT4 was lower, and the translocation of GLUT4 to the plasma membrane was also altered [21]. Ultimately, the decrease in GLUT4 and caveolin-1 associated with atorvastatin results in decreased glucose uptake coupled with increased insulin resistance in adipose tissue. A decrease in glucose uptake is also noted in myocytes and hepatocytes, possibly due to the reduced translocation of GLUT4, leading to hyperglycemia and hyperinsulinemia [17,20-21]. Both hyperglycemia and hyperinsulinemia are noted as early markers of non-insulin-dependent diabetes mellitus.

Insulin receptor substrate-1 (IRS-1) is critical for insulin signaling and is phosphorylated in response to either insulin or insulin-like growth factor (IGF) binding to the insulin receptor (IR) [17]. Through the phosphatidylinositol 3-kinase (P13K) pathway, Akt, also known as protein kinase B, becomes phosphorylated and mediates glucose uptake by controlling GLUT4 translocation to the plasma membrane [17,21]. Takaguri et al. found that atorvastatin treatment decreased the phosphorylation of IRS-1 and Akt, specifically in a dose-dependent manner [21]. RhoA and Rab4 are small G proteins involved in insulin signal transduction via modification of IRS-1 and Akt phosphorylation [17,21]. The Rab4 G-protein is present on GLUT4 vesicles and is involved in the intracellular transportation of GLUT4 to the plasma membrane surface in response to the insulin signals. Due to Rab4's unique properties, it is understood that Rab4 is crucial for insulin-stimulated glucose transport. Takaguri et al. have suggested that a loss of Rab4 function partially mediates a decrease in GLUT4 expression by atorvastatin [21]. As such, it can be suggested that statins contribute to new-onset diabetes by reducing insulin signal transduction and inhibiting necessary phosphorylation events via altering the cellular distribution of small G proteins.

Pancreatic β-cells secrete insulin when there is an increase in intracellular Ca2+ concentration, which is controlled through the opening of voltage-gated calcium channels. Yada et al. have shown that simvastatin inhibited glucose-induced Ca2+ signaling in rat pancreatic islet β-cells by directly blocking L-type Ca2+ channels [19]. The action of simvastatin on calcium channels was immediate, implying a direct interaction rather than an indirect effect due to cholesterol depletion [17]. Alternatively, pravastatin had no effect at clinical doses, but after 24-hour incubation in a high dose, β-cells exhibited a mild decrease in insulin release [17,19]. Therefore, while pravastatin may not directly block the L-type Ca2+ channels, it may impair the Ca2+ channels instead at higher doses. Furthermore, using NB598, a potent and competitive squalene epoxidase inhibitor that blocks the final step of cholesterol synthesis, Xia et al. showed that the chronic depletion of cholesterol impaired insulin secretion and inhibited Ca2+ channel currents of isolated mouse pancreatic islet cells [17,22]. NNB598 may also be referred to as maleate. The overall impact of statins is long-term cholesterol reduction. As such, it has been hypothesized that chronic cholesterol depletion by statins could result in a similar reduction of insulin secretion as NB598 treatment [17]. There is no precise proposed mechanism for the relationship between the inhibition of cholesterol synthesis and impaired Ca2+ channel function. However, it has been suggested that these effects might be due to incorrect sorting of membrane lipid-raft bound proteins or due to changes in the conformation of the Ca2+ channel subunits [22]. 

Although significant research has been conducted in the realm of statins' increasing the risk of new-onset diabetes, the risk varies depending on the different types and doses of statins. The largest and most recent meta-analysis, which included 113,394 patients, was conducted to assess different types and doses of statins. The meta-analysis found that the association of any statin and at any dose with new-onset diabetes incidence remains insignificant [17,23]. Specifically, pravastatin at 40 mg/day was associated with the lowest rate of new-onset diabetes. In contrast, rosuvastatin at 20 mg/day was associated with the highest numeric incidence of diabetes, and the risk was intermediate with atorvastatin at 80 mg/day [17,22]. These differences conclude that the risk of new-onset diabetes depends on the type and dose of statin. There may be underlying risk factors that can contribute to an individual’s susceptibility to developing statin-induced diabetes; however, these precipitating risk factors may vary from patient to patient.

Patients on statin therapy tend to have increased contact with healthcare providers, which may aid in obtaining an earlier diabetes diagnosis. Increased contact with healthcare providers may call for an earlier diabetes diagnosis as routine examinations occur at more regularly scheduled intervals than in individuals who may not be on statin therapy [17]. Though the evidence surrounding statins causing diabetes is conflicting, the more extensive cardiovascular benefits tend to outweigh the potential risk of new-onset diabetes. To avoid the risk of new-onset diabetes, patients should be checked for any pre-existing comorbidities that may hasten the diagnosis. Patients should also be checked for the possibility of familial onset diabetes, amongst other risk factors that may precipitate the onset of diabetes, with statin therapy.

Musculoskeletal damage

**Figure 5:**
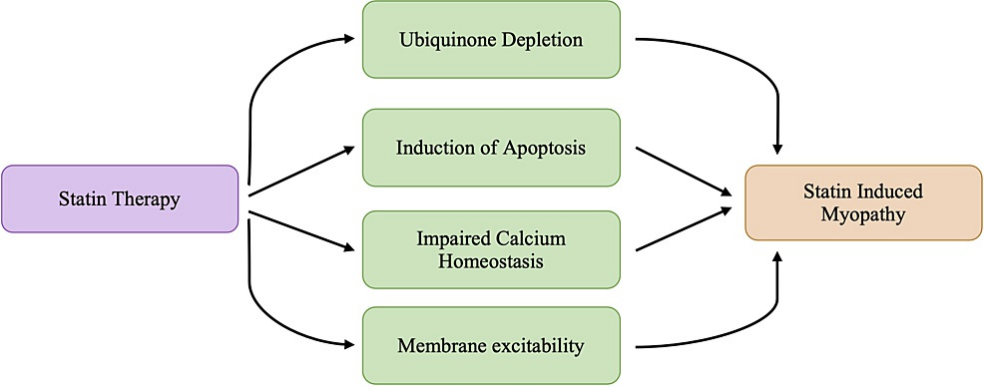
Proposed mechanisms of statin-induced myopathy

Nearly all statin drugs are associated with various musculoskeletal side effects, with myalgia being the most common symptom. Clinical features of statin-induced myopathy typically begin within the first 6-12 months after initiation of therapy. Clinical presentation of statin-induced myopathy includes symptoms such as muscle aches or myalgia, weakness, stiffness, and cramps [8]. Statin-induced myopathy has a highly variable presentation that ranges from muscle tenderness, cramping and muscle aches, weakness, and increased creatine kinase (CK) levels [24]. Evidence indicates that statins can cause self-limited myotoxicity, presumably due to the direct effect of statins on the muscle or an autoimmune myopathy associated with autoantibodies targeting HMG-CoA reductase [7]. Though the exact pathological mechanisms underlying musculoskeletal damage are not understood, each is a proposed mechanism caused by a decrease in various components because of a blockade early in the mevalonate pathway, as shown in Figure 1. A decrease in the concentration of the various byproducts calls for a different mechanism leading to the statin-induced myopathy that may be seen in some patients. The most identified risk factors for statin-associated muscle complaints are associated with high-dose exposure, advanced age, female gender, small body frame, and multisystem diseases [24]. Figure 5 lays out commonly proposed mechanisms leading to myositis and other myopathies witnessed in statin therapy.

Despite all the proposed mechanisms, it is crucial to understand that the exact pathophysiology of what leads to the presentation of statin-induced myopathies is still not fully understood. The presentation of statin-induced myopathy differs from patient to patient, and this may be due to any number of factors. The most important risk factors for statin-induced myopathies are advanced age, female gender, Asian ethnicity, drugs altering statin plasma levels, excessive physical activity, muscle, liver, or chronic kidney diseases, uncontrolled hypothyroidism, abdominal obesity, metabolic syndrome, and vitamin D deficiency [24-25]. Each risk factor increases the vulnerability to statin-induced adverse effects as they decrease the individual’s immune response, causing the susceptibility to adverse effects to increase. While several different pathophysiological pathways have been associated with statin-induced myopathy, each pathway proposed in Figure 5 can be explained concomitantly with other proposed theories. Current research does not outline a unified mechanism as the overall cause of statin-induced myopathy, but each proposed mechanism explains the shortcomings of other proposed theories. This can be primarily due to the notion that statins not only reduce cholesterol levels in the body but also impact other byproducts.

Statin-induced myopathy is more likely to occur with more potent statins or at higher doses. A recent study evaluated the incidence of myopathy in patients under statin and concluded that the incidence of statin-induced myopathy was highest with simvastatin and lowest with fluvastatin and rosuvastatin [8]. Variations between each statin’s physicochemical structure have provided clues in some patients experiencing myopathy, which allows the ability to switch statins without continued pain [5]. It has been postulated that both atorvastatin and rosuvastatin are good choices in order to reduce the occurrence of statin myopathy because of their long half-lives, which enable an alternate-day or twice-weekly dosing strategy [5,8]. In the more extreme cases, myopathy can take the form of rhabdomyolysis, a condition in which patients have CK levels > 10,000 IU/L [5,13]. Rhabdomyolysis is a pathological syndrome in which muscle degeneration occurs and the circulatory system becomes infested with intracellular species such as myoglobin [25]. Elevated creatine phosphokinase (CPK) levels are diagnostic of rhabdomyolysis. The excess myoglobin in the circulatory system can lead to glomerular failure, ultimately impacting the kidneys and possibly the liver [13]. While rhabdomyolysis is the most well-known adverse effect of statin use, it is relatively rare, occurring in less than 0.1% of statin users [13,25]. Due to its extreme rarity in occurrence, rhabdomyolysis does not compromise the entirety of the muscle-related complaints amongst patients on statins. However, less-severe, non-rhabdomyolysis-related muscle complaints have been shown to occur with a higher frequency [13]. A characteristic physical symptom of rhabdomyolysis is a case of severe myalgia and dark urine [25]. The dark urine is indicative of the presence of excess myoglobin in the urine. It is typically monitored in patients on a high dose of simvastatin in the early phase of the medication's initiation [13]. Furthermore, the risk of rhabdomyolysis has been noted to be significantly increased when other drugs such as fibrates, cyclosporines, macrolide antibiotics, and azole antifungals are concomitantly administered [13,25]. While Figure 5 lists different proposed mechanisms that may explain myopathies associated with statin use, the overall pathophysiology of statin-induced myopathy can be attributed to a combination of the different mechanisms listed.

Decreased production of ubiquinone CoQ10 may result in muscle damage. Ubiquinone is a key protein in charge of stabilizing the cell membrane and is also known to play a crucial role in the mitochondrial respiratory chain [7,26]. Furthermore, CoQ10 prevents oxidative stress and regenerates active antioxidant vitamins C and E [27]. CoQ10 is typically found in cholesterol-rich foods. The depletion of CoQ10 in myocyte mitochondria may disturb cellular respiration and consequently cause muscle-related toxicities, including rhabdomyolysis [28]. Decreased production of CoQ10 further impairs the mitochondrial function of myocytes, resulting in an increased presentation of musculoskeletal symptoms. Biopsies of patients with statin-induced myopathy have been noted to show an unusual accumulation of lipids, which is consistent with a decrease in mitochondria-mediated oxidative metabolism [24]. Statin-induced myopathy can be prevented in patients by lowering the dosage of statin therapy just enough to achieve therapeutic goals. Achieving the appropriate therapeutic dose that decreases the likelihood of statin-induced myopathies among individuals may be a process of trial and error for physicians. Still, it can increase the likelihood of continuing the statin regimen.

Apoptosis is a type of programmed cell death that is regulated and executed through various signaling pathways. It has been shown that statins can induce apoptosis in various cell types, such as rheumatoid synovial cells, pericytes, smooth muscle cells, cardiac myocytes, and several types of cancer cells [28-29]. The apoptosis of these cells mentioned above in diseased tissue may contribute to the pleiotropic benefits of statins [29]. The notion that a compound possesses pleiotropic properties suggests that the compound can produce more than one effect. In the case of statins, there is evidence that suggests that some of the benefits of statin therapy can be mediated by their pleiotropic properties, which include stabilization of atherosclerotic plaques, anti-inflammatory properties, improvement of endothelial dysfunction, and anti-thrombotic effects [8]. In diseased cells, the pleiotropic effects of statins may be beneficial in stabilizing the cells and decreasing any adverse conditions from developing. However, in healthy myocytes, statin-induced apoptosis may be the contributing factor causing myopathy. It has been shown that the depletion of isoprenoids is responsible for the induction of apoptosis [29].

Isoprenoids are one of the end products of cholesterol synthesis and play an essential role in post-translational modifications and in multiple signaling molecules of membrane attachment [28]. Statin therapy would deplete isoprenoids by inhibiting the enzyme HMG-CoA reductase. Experiments show that inhibiting squalene synthase, an enzyme downstream of HMG-CoA reductase in cholesterol biosynthesis, does not cause myotoxicity [28-29]. This evidence suggests that statin-induced apoptosis is not due to a decreased cholesterol concentration but rather to the suppressed synthesis of the alternative products of the mevalonate pathway. Isoprenoids serve as important intermediate lipid moieties for posttranslational modifications of membrane-associated proteins such as Ras, Rho, and Rac [28]. Ras is a small G protein that regulates cellular proliferation, differentiation, and transformation [28-30]. Rho and Rac are also members of the Rho family of small G proteins [29]. Isoprenylation, farnesylation, and geranylgeranylation of these proteins regulate their translocation to the plasma membrane [29-30]. For example, farnesylation of Ras is required for its translocation from the cytoplasm to the plasma membrane, where it becomes activated [29-30]. Farnesylation of the Ras protein is crucial for proper signaling function [30]. Geranylgeranylation of Rho is required for its translocation to the plasma membrane [29]. Depleted isoprenoids lead to decreased protein geranylgeranylation and/or farnesylation, causing elevated cytosolic Ca2+ and activation of mitochondrial-mediated apoptotic signaling cascade [28]. Blockade within the mevalonate pathway reduces farnesyl and geranylgeranyl residues required for the appropriate attachment of different small G proteins to the cellular membrane [29-30]. The appropriate attachment of small G proteins to the cellular membranes modulates the immune response at different levels, including T-cell signaling, antigen presentation, immune cell migration, and cytokine production [30]. In vitro studies concluded that lovastatin inhibited T-cell proliferation, Ca2+ influx, and T cells' interleukin-2 (IL-2) production [29]. Alongside the more direct effects due to the reduction of isoprenylation, statins also cause other cellular changes. Statins impact the gene expression of pro-inflammatory genes in the innate and adaptive immune systems and also in non-hemopoietic cells, including endothelial cells and fibroblasts [30]. As statins impact endothelial cells and fibroblasts, another possible mechanism explaining statin myopathy could be the result of a reduction in the isoprenoids involved in muscle fiber apoptosis [29]. Apoptosis of myofibers due to a reduced level of isoprenoids could explain why larger muscle groups such as the thighs, buttocks, calves, and back muscles tend to be the characteristic sites of muscle weakness and muscle pain [29-31].

Animal models have shown that statins decrease strength and increase cytosolic Ca2+ by elevating both mitochondrial Ca2+ permeability and Ca2+ release from the sarcoplasmic reticulum (SR) via the ryanodine receptors [28,31]. Typically, Ca2+ is only released into the rest of the cell during muscular contractions, and the release of Ca2+ is modulated by a type of gatekeeper protein known as the FK506 binding protein [32]. The FK506-binding protein stabilizes the closed state of the ryanodine receptor to ensure there is no leakage of Ca2+ in the absence of muscular contractions. It is postulated that statins will cause the FK506-binding protein to dissociate from the ryanodine receptor. When this occurs, the ryanodine receptors become destabilized in their closed state. As such, it is postulated that Ca2+ will continuously leak into the cell even when muscles are at rest [31-32]. Based on muscle biopsies, researchers have found a link between Ca2+ leakage and muscle cell damage, leading to symptoms of myalgia or cramps [28]. It could be assumed that if Ca2+ leakage is the reason for muscle pain and weakness, every patient on statin therapy should be experiencing these muscular adverse effects. However, some patients may probably be more susceptible due to their genetic predisposition or lifestyles; thus, the calcium leakage strains their already-burdened physiological homeostasis.

Cholesterol also modulates membrane fluidity in various tissues, including skeletal muscles [8,28]. A decreased cholesterol level causes the membrane to become more rigid and, in doing so, can impair the function of ion channels and, consequently, ion transport. Altered membrane fluidity will modify membrane excitability by impairing the sodium, potassium, and chloride ion channels [28]. Chloride channels play an essential role by controlling the resting membrane potential and membrane depolarization in skeletal muscles [33]. Impaired Cl^−^ ion channel function can impair membrane depolarization and alter the resting membrane potential. This suggests that affected cells may not be able to respond to stimulus as visible at the normal resting membrane potential. Chronic simvastatin treatment in rabbits has been shown to trigger membrane hyperexcitability, which was previously observed in muscle myopathies associated with impaired chloride conductance [28,33].

Hepatic dysfunction

**Figure 6:**
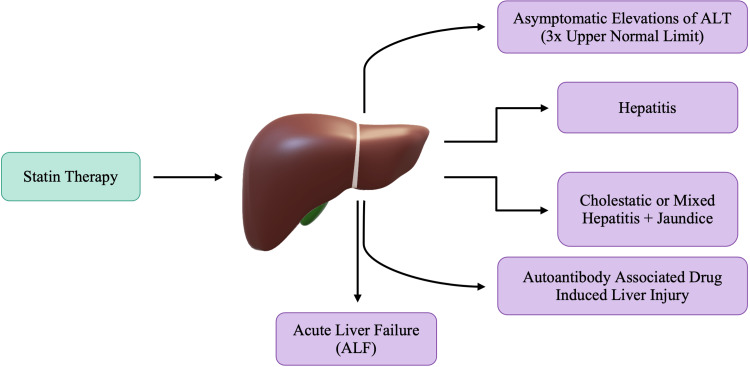
Proposed adverse effects of statin-induced hepatic dysfunction

Hepatotoxicity is defined as liver damage directly resulting from drugs or other chemicals [34]. Clinical studies on statins have demonstrated a 0.5-3.0% occurrence of persistent elevations in aminotransferases in patients on statin therapy [8]. The probability of statin-induced hepatic dysfunction increases when statins are administered at their maximum doses or when statins are used in conjunction with other lipid-lowering drugs. Hepatocytes are considerable contributors to cholesterol production and, therefore, are a significant target cell of statins. As detailed previously, cholesterol allows cellular membranes to maintain their fluidity, so ions and transport channels can function correctly. As statins cause a decrease in cholesterol content, cell membranes lose their fluidity, making them more rigid and fragile. Furthermore, the rigid cellular membrane will cause leakage of intracellular contents and, in doing so, cause an elevation in transaminase levels. Hepatic adverse effects in response to statin administration were initially defined as alanine aminotransferase (ALT) being as high as 3 times the upper limit of normal, which was observed in up to 3% of patients [35]. The phenomenon is not specific to statins, as many other drugs also cause an asymptomatic elevation in liver enzymes. This transient elevation in ALT is not well understood but has been called adaptation [36]. Clinically, drug-induced liver injury is infrequent with statin use. Figure 6 outlines that acute liver failure (ALF) has also been noted with statin therapy; however, ALF develops in a very small minority of patients taking statins [34,36]. The risk of developing ALF in response to statin therapy is very minimal, and the incidence was not much different from that in the general population [36].

As mentioned previously, there may be an asymptomatic elevation of ALT; however, some patients may also present with clinical symptoms of jaundice, therefore confirming the diagnosis of hepatitis. Autoimmune hepatitis may also be noted in the presence of antinuclear antibody (ANA), anti-smooth muscle antibody, or antimitochondrial antibody with or without plasma cells on liver biopsy [36]. Recent studies show that autoimmune hepatitis generally occurs two to seven months after statin initiation and can persist even after the statin therapy is discontinued [35]. Despite the concerns about statin-induced hepatotoxicity, the clinically relevant risk of liver damage from statins remains considerably low in patients without cirrhosis. Therefore, it should not outweigh the broad cardiovascular benefits [35-36].

Mitochondrial dysfunction

**Figure 7:**
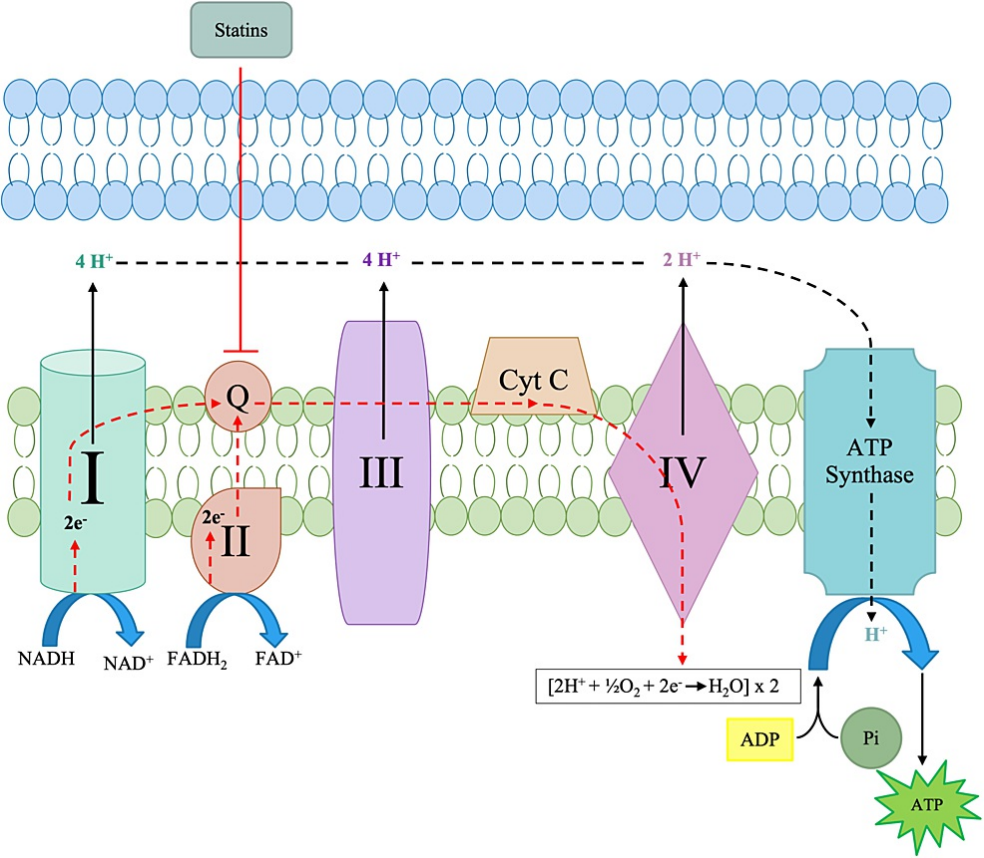
Statins' site of inhibition in the electron transport chain

Considered the cell's powerhouse, the mitochondria are organelles in eukaryotic cells. Several cellular respiration pathways occur within the mitochondria, including the Krebs cycle, oxidative phosphorylation, and fatty acid b oxidation [37]. Oxidative phosphorylation is critical and is carried out by several protein complexes and molecules on the inner mitochondrial membrane, as illustrated in Figure 6. The oxidative phosphorylation pathway consists of two subsequent steps: the electron transport chain and adenosine 5’ triphosphate (ATP) synthesis [38]. ATP synthesis is the last step in the oxidative phosphorylation pathway. ATP synthesis is the phosphorylation of ADP to ATP catalyzed by ATP synthase due to the proton motive force following the electrochemical gradient across the inner mitochondrial membrane [37-38]. Each of the complexes shown on the inner mitochondrial membrane in Figure 7 plays a vital role in oxidative phosphorylation. Inhibition or downregulation of any of the complexes results in a decrease in ATP output, which can lead to many symptoms of decreased energy and myalgia, as noted earlier. Mitochondrial dysfunction is typically defined as a decrease in the ability of the mitochondria to synthesize high-energy compounds such as ATP because of a suboptimal electron transfer rate across the respiratory chain complexes [38]. Mitochondrial dysfunction can lead to a wide array of adverse effects; however, it is most commonly noted in statin-induced myopathy.

Mitochondrial dysfunction is defined as a decrease in the ability of the mitochondria to synthesize high-energy compounds such as ATP and a suboptimal transfer rate across the respiratory chain complex [39]. Mitochondrial dysfunction can be precipitated by drugs and is now the most widely accepted and studied pathobiological mechanism for statin-associated muscle symptoms. However, the exact nature and extent of mitochondrial dysfunctions causing statin-associated muscle symptoms remain unclear [37,39]. Statin-induced coenzyme Q10/ubiquinone deficiency is the most common and extensively studied etiological cause of statin-induced myopathies, as denoted in Figure 7 [39].

As described earlier in this article concerning myositis, the effects of statins are central in the mitochondrial electron transport chain, explicitly impacting CoQ10. As with all other adverse effects, the effects of statins on the mitochondria are noted to a greater degree in vulnerable individuals. The effects of statins on the mitochondria and ETC are dose-dependent. Statin use can decrease CoQ10, reducing cell energy, promoting oxidation and apoptosis, and unmasking any silent mitochondrial effects [5]. As illustrated in Figure 7, statins will directly impact the mitochondrial respiratory chain and specifically impact CoQ10. CoQ10 is a vital electron transporter in the mitochondrial respiratory chain, and a decrease in this compound could result in abnormal mitochondrial respiratory function, causing mitochondrial and oxidative damage [40-41]. Alongside a decrease in CoQ10, higher lactate/pyruvate ratios are also noticed, and both indicate abnormal mitochondrial function [37]. The cholesterol biosynthesis blockade has been linked to lowering mitochondrial membrane potential and increasing the release of pro-apoptotic factors [42]. While not actively noted in current research, lowering the mitochondrial membrane potential can also cause hyperexcitability.

Significant alterations in the mitochondrial membrane potential can cause cell death [40]. Mitochondrial dysfunction is most notable in the skeletal muscles through the presentation of myositis and other myopathies. Phillips et al. noted three patients who exhibited symptoms of myopathy likely related to statin use. Muscle biopsies showed abnormally elevated lipid stores, ragged red fibers, and fibers with decreased staining for cytochrome c activity, a biomarker of mitochondrial activity [43]. These abnormal findings have been noted to disappear after the cessation of statin therapy. Furthermore, comparing the treatment of 80 mg/day simvastatin with that of 40 mg/day atorvastatin for eight weeks showed a 30% decrease in muscle CoQ10 in the simvastatin group, along with decreased activity of ETC enzymes [37]. In vitro, functional impairment of complexes I, III, and IV occurred in rat skeletal muscles after 24-hour exposure to 100 μmol/L cerivastatin, simvastatin, fluvastatin, or atorvastatin [38].

Mitochondrial dysfunction is typically linked with intrinsic apoptosis, also known as the mitochondrial apoptotic pathway. This pathway is characterized by the activation of caspase 3 and caspase 9, and the activation or inhibition of pro-apoptotic BCL-2 or anti-apoptotic BL2 family members [5,40]. Statin-induced mitochondrial dysfunction could be due to any combination of factors. The intense oxidative stress that induces mitochondrial dysfunction can be attributed to statin-induced decreases in CoQ10 and dolichol levels, which are considered antioxidant defense systems. Statins inhibit the mitochondrial respiratory chain's functioning and uncouple oxidative phosphorylation, causing a reduction in the membrane potential [39]. A decrease in the mitochondrial membrane potential may result in increased permeability of the inner mitochondrial membrane, which is believed to initiate programmed cell death [44]. This increase in membrane permeability can lead to a leaky outer membrane, thus increasing the volume of the mitochondrial matrix (mitochondrial swelling) and the outflow of cytochrome c from the intermembrane space to the cytosol [39,44]. Despite all these proposed mechanisms, the exact effect of statins on the mitochondria of human cells is still not fully understood, and further studies are deemed necessary. Further research and studies can allow for an in-depth understanding of the effects of statins on the mitochondria and their association with statin-induced myopathies.

Neurocognitive effects

The human brain contains approximately 25% of the total cholesterol in the body [45]. For the most part, cholesterol in the adult brain is largely metabolically inert, with an estimated 0.02% undergoing turnover daily [46]. Unlike cholesterol in the plasma, which has a half-life of only a few days, brain cholesterol has been associated with a half-life that ranges from six months to five years [45]. The extremely long half-life of cholesterol in the brain suggests that chronic therapy may be required for any statin-induced neurological effects to present.

Cholesterol contributes to the myelin sheath and the membrane lipid rafts noted in neurons and astrocytes, regulating ion channel permeability, signal transmission, and other cellular functions [45-46]. Abnormalities in cholesterol metabolism in the central nervous system (CNS) can be associated with many neurocognitive effects. Statins may reduce cholesterol synthesis in the brain and interfere with myelin formation and function [45]. Neurons in the brain are surrounded by supporting cells called glia. Glial cells are responsible for repairing damaged neurons. These glial cells also produce a fatty cholesterol coating to insulate the neurons. Statins block these glial cells from producing cholesterol, which can cause cognitive impairment [47]. Though not thoroughly researched, it has been hypothesized that statins have several short-term and long-term effects on cognitive and neurological function, which can be attributed to the physicochemical properties of various compounds. The effects of statins on cognitive and neurological function can be attributed to the physicochemical properties of the various compounds. It has been hypothesized that due to the increased permeability of lipophilic statins, most of the neurological effects may be due to lipophilic statins; however, new research suggests that hydrophilic statins can also enter the neuroparenchyma [47-48].

**Figure 8:**
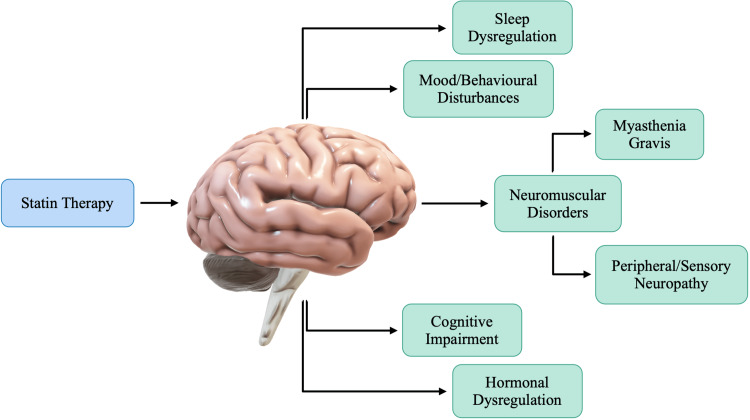
Proposed neurological adverse effects of statin therapy

The proposed mechanisms of statins' effects on an individual's neurocognition have been outlined in Figure 8. It is imperative to understand that, whilst many mechanisms have been proposed, the true effects of statins on neurocognition require more research and understanding. Whether the effects on cognitive function are associated with statins directly or due to decreased total cholesterol levels is yet to be understood. Guo et al. conducted a study that focused on the effects that statins may have on the brain, specifically the hippocampus. Data collected and presented by Guo et al. provide several lines of evidence that BBB-permeable simvastatin may impair cognition by reducing hippocampal cholesterol [48]. Figure 8 also mentions peripheral neuropathy as a neurological statin-induced adverse effect. The incidence of polyneuropathy has been reported more frequently with atorvastatin than with fluvastatin [48-49]. Furthermore, the duration of statin therapy has been noted as a significant risk factor for developing sensory neuropathy [48]. As mentioned previously, cholesterol plays a vital role in the myelination of nerve fibers in the central and peripheral nervous systems. As such, statins may lead to peripheral nerve alterations with axonal involvement but without any clinical manifestations [49]. While some patients may not present with clinical manifestations, the symptomatic presentation may be much more significant for others. This can be largely attributed to any comorbidities, along with risk factors. While neuropathy has been noted, there is no conclusive evidence for a causal relationship between statin treatment and neuropathies, and due to this, further research is required.

Statin therapy has also been noted to cause mood disturbances, although the precise mechanisms remain unclear. A case series conducted by Cham et al. found behavioral and psychiatric changes that ranged from violent nightmares to aggression, mood/personality changes, and violent or homicidal ideation, each in apparent association with statin use [49]. The association between the drug initiation and the behavioral and mood changes that would be resolved upon statin cessation suggests a causal connection in several cases [49-50]. Statins have been associated with mitochondrial dysfunction, and mitochondrial dysfunction has been documented in a range of psychiatric problems and, as such, may also contribute to behavioral change with statin-induced oxidative damage [49]. The actual effects of statins on neurocognitive function can be disputed as research continues to emerge in this field. Many factors must be considered in patients presenting with any behavioral or mood changes. A patient’s vulnerability to aggression or any other psychiatric changes must be noted prior to attributing any such adverse effects to statin therapy.

Another very sporadic side effect of statins is the possible induction of autoimmune illnesses such as myasthenia gravis (MG). MG is an autoimmune disorder that is characterized by muscle weakness due to an altered transmission at the neuromuscular junction and autoantibodies against the acetylcholine receptors (AChRAb) [50]. The association between MG and statins remains unclear; however, it has been postulated that it may be due to the immunomodulatory effects of statins on T and B cells [4,50]. In humans, statins may inhibit human B-lymphocyte activation and major histocompatibility complex II (MHC II) presentation [4]. This inhibition may lead to an increase in antibody-mediated humoral immunity [4,50]. MG is an antibody-mediated disease caused by autoantibodies against the acetylcholine receptors at neuromuscular junctions. An autoantibody is an antibody produced by the immune system and directed against one or more of the individual’s own proteins. The increase of antibody-mediated humoral immunity due to statins may be the prime reason why myasthenic symptoms may worsen in patients on statin therapy. Because of the rarity of the occurrence of developing MG as an adverse effect, patients with dyslipidemia and myasthenia gravis should continue statin therapy. However, it should be the physician’s responsibility to inform the patient that statins could worsen myasthenic symptoms and to be watchful for any deteriorating signs.

Various clinical studies and meta-analyses were utilized throughout this manuscript to understand the adverse effects of statins. Table 1 provides a summarization of the various studies and their key findings. Further clinical studies must be conducted to explore the adverse effects of statin therapy. Many studies provide the same conclusory results, suggesting some connection between statins and these adverse effects. Statin administration by healthcare providers will continue as a primary and secondary prevention of cardiovascular diseases; however, patients must be aware of the side effects. Vulnerable patients, such as those with comorbidities, are much more likely to present with adverse effects; however, further research must be conducted in the field to understand if there is a likely connection. Clinical studies noted in Table 1 could not conclude if vulnerable patients were more likely to present with adverse effects. Furthermore, mitochondrial dysfunction and oxidative stress were noted by many of the studies listed in Table 1 as being a probable mechanism for many of the effects.

**Table 1:** Summarization of clinical trials

Author	Publication year	Participants/cell cultures	Treatment	Results
Pinal-Fernandez et al. [7]	2018	Meta-analysis of randomized trials – 5 trials; 39,612 individuals	Statin therapy: less intensive statin therapy versus more intensive regimens	LDL cholesterol decreased by 2 mmol/L at 5 years in 10,000 patients reduced rate of major vascular events by 10% in secondary prevention and 5% in primary prevention
Jackevicius et al. [11]	2002	Patients aged 66 years or older who received at least 1 statin prescription between January 1994 and December 1998	Comparing adherence to statins in 3 cohorts: those with acute coronary syndrome, chronic coronary artery disease, and those without coronary disease (primary prevention).	2-year adherence rates were only 40.1% for acute coronary syndrome, 36.1% for chronic coronary artery disease, and 25.4% for primary prevention.
Sattar et al. [16]	2010	91,140 patients (mean age range 55 to 76 with BMI range from 24 to 29 kg/m^2^), with a risk/history of cardiovascular disease	Atorvastatin, lovastatin, rosuvastatin, and simvastatin were studied at varying doses	Statin therapy was associated with a slightly increased risk of developing diabetes
Carter et al. [18]	2013	Patients aged 66 years or older without diabetes who started statin therapy from August 1, 1997, to March 31, 2010	Statin therapy	Increased risk of diabetes with atorvastatin, rosuvastatin, and simvastatin. No increase in risk among those who received fluvastatin.
Nakata et al. [19]	2006	3T3L1 adipocytes (cell line of adipocytes specifically used to study adipogenesis)	Clinical doses of atorvastatin for 15 weeks	Decreased expression of GLUT4
Yada et al. [20]	1999	Islet and single β-cells isolated from Wistar rats aged 8-12 weeks	Simvastatin, simvastatin (simvastatin-acid), and pravastatin. Simvastatin was dissolved in 100% ethanol and simvastatin-acid and pravastatin were distilled in water.	Simvastatin inhibited glucose-induced Ca^2+^ signaling in rat pancreatic islet β​​​​​​​-cells by directly blocking L-type Ca^2+^ channels
Takaguri et al. [21]	2008	3T3LI fibroblasts were obtained from a Health Science Research Resources Bank in Osaka	Treatment with atorvastatin and pravastatin	Treatment with 10 μM atorvastatin for 72 hours decreased insulin-stimulated 2-deoxy-glucose uptake. Same results were not noted with pravastatin treatment.
Xia et al. [22]	2008	Mouse pancreatic islet cells	Isolated islet cells cultured in the absence and presence of different doses of NB598 (cholesterol biosynthesis inhibitor)	Decrease in cholesterol impairs insulin secretion
Navarese et al. [23]	2013	Meta-analysis of 13 randomized controlled trials – 113,394 patients	Different types and different doses of statin and association with new-onset diabetes	Pravastatin therapy is associated with the lowest rate of new-onset diabetes. Rosuvastatin was associated with the highest incidence of diabetes. High-dose pravastatin safest treatment in terms of new-onset diabetes.
Ghirlanda et al. [26]	1993	Group A: 10 healthy volunteers (age 34.2 ± 8 years, male, BMI 23.1 ± 0.6 kg/m^2^); group B: 30 hypercholesteremic patients matched for age and sex	Group A: 20 mg/daily of pravastatin or simvastatin for 1 month at bed Group B: 10 patients treated with 20 mg/day pravastatin, 10 patients 20 mg/day simvastatin, 10 patients given a placebo for 3 months at bedtime	Simvastatin and pravastatin were well tolerated by all groups. Body weight remained unchanged.
Lorkowska et al. [31]	2004	Cultured bovine aortic endothelial cells	Simvastatin, atorvastatin, pravastatin, and cerivastatin administration in a concentration range of 10-30 mM	Decrease in strength and increase in cytosolic Ca^2+^ by elevating mitochondrial Ca^2+^ permeability and Ca^2+^ release from SR
Phillips et al. [43]	2002	4 patients with muscle symptoms developed during statin therapy	Muscle biopsy and creatine kinase levels measured	3 patients’ muscle biopsies showed abnormally elevated lipid stores, ragged red fibers, and decreased staining for cytochrome c activity
Guo et al. [48]	2021	Animal study conducted on mice	Administration of simvastatin for 26 consecutive days	Blood-brain barrier permeable simvastatin may impair cognition by reducing hippocampal cholesterol
Cham et al. [49]	2016	12 subjects and/or family members who experienced mood and behavioral changes while on statin therapy	Survey information provided	According to survey information, behavioral and psychiatric changes ranged from violent nightmares to aggression, and mood/personality changes, all in apparent statin use

## Conclusions

HMG-CoA reductase inhibitors, also referred to as statins, have played a pivotal role in lowering LDL cholesterol, thus decreasing the chances of any cardiovascular diseases. In addition, compared with placebos, statins raise both HDL and apolipoprotein A-1, and these elevations are maintained in the long term. Furthermore, statins also decrease LDL and triglycerides. However, statins present with their own unique panel of adverse effects that is of utmost clinical significance. This review, for the first time, has attempted to focus on statin-induced diabetes. In many cases, the benefits of statin therapy outweigh the risk of adverse effects. However, in a smaller patient population, the adverse effects may be severe enough to cause healthcare providers to consider discontinuation of the drug altogether. The physicochemical properties of statins explain why some are more likely to cause detrimental side effects than others. Lipophilic statins are considered more membrane permeable and, as such, are more likely to lead to adverse effects. The lipophilicity of statins allows for their passage through the BBB, leading to the presentation of neurocognitive effects such as cognitive impairment. While the evidence suggesting this claim is relatively weak, the permeability of statins can also be attributed to mitochondrial dysfunction and statin-induced myopathies. Understanding the correct dosage and the most appropriate statin compound for each patient is crucial to ensuring that the panel of potential adverse effects is controlled. Co-existing conditions must also be considered before statin therapy, and patients should be monitored throughout the use of statins. More research needs to be conducted on the adverse effects of statins, as the range of effects is too diverse. Inhibition of HMG-CoA reductase decreases the body's total cholesterol levels and the concentration of many other products of the cholesterol synthesis pathway. Understanding the effects of each of these products on the cholesterol pathway allows for an understanding of the adverse effects that a decrease in the products could cause. Current research shows that the benefits of statin therapy significantly outweigh the risks of its adverse effects. However, further research will allow healthcare practitioners to understand whether primary prevention with statin therapy could be necessary for some patients.

## References

[1] (2022). Heart disease facts. https://www.cdc.gov/heartdisease/facts.htm.

[2] (2022). Heart disease and stroke. https://www.cdc.gov/chronicdisease/resources/publications/factsheets/heart-disease-stroke.htm.

[3] (2022). HDL (good), LDL (bad) cholesterol and triglycerides. https://www.heart.org/en/health-topics/cholesterol/hdl-good-ldl-bad-cholesterol-and-triglycerides.

[4] Ramkumar S, Raghunath A, Raghunath S (2016). Statin therapy: Review of safety and potential side effects. Acta Cardiol Sin.

[5] Golomb BA, Evans MA (2008). Statin adverse effects: a review of the literature and evidence for a mitochondrial mechanism. Am J Cardiovasc Drugs.

[6] Buhaescu I, Izzedine H (2007). Mevalonate pathway: a review of clinical and therapeutical implications. Clin Biochem.

[7] Pinal-Fernandez I, Casal-Dominguez M, Mammen AL (2018). Statins: pros and cons. Med Clin (Barc).

[8] Egom EE, Hafeez H (2016). Biochemistry of statins. Adv Clin Chem.

[9] Go AS, Mozaffarian D, Roger VL (2013). Executive summary: heart disease and stroke statistics--2013 update: a report from the American Heart Association. Circulation.

[10] Istvan ES (2002). Structural mechanism for statin inhibition of 3-hydroxy-3-methylglutaryl coenzyme A reductase. Am Heart J.

[11] Jackevicius CA, Mamdani M, Tu JV (2002). Adherence with statin therapy in elderly patients with and without acute coronary syndromes. JAMA.

[12] DuBroff R, de Lorgeril M (2015). Cholesterol confusion and statin controversy. World J Cardiol.

[13] Azemawah V, Movahed MR, Centuori P (2019). State of the art comprehensive review of individual statins, their differences, pharmacology, and clinical implications. Cardiovasc Drugs Ther.

[14] Climent E, Benaiges D, Pedro-Botet J (2021). Hydrophilic or lipophilic statins?. Front Cardiovasc Med.

[15] Althanoon Z, Faisal IM, Ahmad AA (2020). Pharmacological aspects of statins are relevant to their structural and physicochemical properties. Sys Rev Pharm.

[16] Sattar N, Preiss D, Murray HM (2010). Statins and risk of incident diabetes: a collaborative meta-analysis of randomised statin trials. Lancet.

[17] Brault M, Ray J, Gomez YH, Mantzoros CS, Daskalopoulou SS (2014). Statin treatment and new-onset diabetes: a review of proposed mechanisms. Metabolism.

[18] Carter AA, Gomes T, Camacho X, Juurlink DN, Shah BR, Mamdani MM (2013). Risk of incident diabetes among patients treated with statins: population based study. BMJ.

[19] Nakata M, Nagasaka S, Kusaka I, Matsuoka H, Ishibashi S, Yada T (2006). Effects of statins on the adipocyte maturation and expression of glucose transporter 4 (SLC2A4): implications in glycaemic control. Diabetologia.

[20] Yada T, Nakata M, Shiraishi T, Kakei M (1999). Inhibition by simvastatin, but not pravastatin, of glucose-induced cytosolic Ca2+ signalling and insulin secretion due to blockade of L-type Ca2+ channels in rat islet beta-cells. Br J Pharmacol.

[21] Takaguri A, Satoh K, Itagaki M, Tokumitsu Y, Ichihara K (2008). Effects of atorvastatin and pravastatin on signal transduction related to glucose uptake in 3T3L1 adipocytes. J Pharmacol Sci.

[22] Xia F, Xie L, Mihic A, Gao X, Chen Y, Gaisano HY, Tsushima RG (2008). Inhibition of cholesterol biosynthesis impairs insulin secretion and voltage-gated calcium channel function in pancreatic beta-cells. Endocrinology.

[23] Navarese EP, Buffon A, Andreotti F (2013). Meta-analysis of impact of different types and doses of statins on new-onset diabetes mellitus. Am J Cardiol.

[24] Sewright KA, Clarkson PM, Thompson PD (2007). Statin myopathy: incidence, risk factors, and pathophysiology. Curr Atheroscler Rep.

[25] Bagley WH, Yang H, Shah KH (2007). Rhabdomyolysis. Intern Emerg Med.

[26] Ghirlanda G, Oradei A, Manto A (1993). Evidence of plasma CoQ10-lowering effect by HMG-CoA reductase inhibitors: a double-blind, placebo-controlled study. J Clin Pharmacol.

[27] Joy TR, Hegele RA (2009). Narrative review: statin-related myopathy. Ann Intern Med.

[28] Tomaszewski M, Stępień KM, Tomaszewska J, Czuczwar SJ (2011). Statin-induced myopathies. Pharmacol Rep.

[29] Dirks AJ, Jones KM (2006). Statin-induced apoptosis and skeletal myopathy. Am J Physiol Cell Physiol.

[30] Laufs U, Liao JK (2003). Isoprenoid metabolism and the pleiotropic effects of statins. Curr Atheroscler Rep.

[31] Lorkowska B, Chlopicki S, Marcinkiewicz E, Gryglewski RJ (2004). Statins rise cytoplasmic calcium level [Ca2+]i in cultured endothelial cells. Pol J Pharmacol.

[32] Chelu MG, Danila CI, Gilman CP, Hamilton SL (2004). Regulation of ryanodine receptors by FK506 binding proteins. Trends Cardiovasc Med.

[33] Sirvent P, Mercier J, Lacampagne A (2008). New insights into mechanisms of statin-associated myotoxicity. Curr Opin Pharmacol.

[34] Cueto R, Valdivielso P, Lucena MI, García-Arias C, Andrade RJ, González-Santos P (2008). Statins: hepatic disease and hepatotoxicity risk. Open Gastroenterol J.

[35] Francis P, Forman L (2020). Use of statins in patients with and without liver disease. Clin Liver Dis (Hoboken).

[36] Thapar M, Russo MW, Bonkovsky HL (2013). Statins and liver injury. Gastroenterol Hepatol (NY).

[37] Apostolopoulou M, Corsini A, Roden M (2015). The role of mitochondria in statin-induced myopathy. Eur J Clin Invest.

[38] Mollazadeh H, Tavana E, Fanni G (2021). Effects of statins on mitochondrial pathways. J Cachexia Sarcopenia Muscle.

[39] Ramachandran R, Wierzbicki AS (2017). Statins, muscle disease and mitochondria. J Clin Med.

[40] Tricarico PM, Crovella S, Celsi F (2015). Mevalonate pathway blockade, mitochondrial dysfunction and autophagy: a possible link. Int J Mol Sci.

[41] Tavintharan S, Ong CN, Jeyaseelan K, Sivakumar M, Lim SC, Sum CF (2007). Reduced mitochondrial coenzyme Q10 levels in HepG2 cells treated with high-dose simvastatin: a possible role in statin-induced hepatotoxicity?. Toxicol Appl Pharmacol.

[42] Tricarico PM, Marcuzzi A, Piscianz E, Monasta L, Crovella S, Kleiner G (2013). Mevalonate kinase deficiency and neuroinflammation: balance between apoptosis and pyroptosis. Int J Mol Sci.

[43] Phillips PS, Haas RH, Bannykh S (2002). Statin-associated myopathy with normal creatine kinase levels. Ann Intern Med.

[44] Broniarek I, Jarmuszkiewicz W (2016). [Statins and mitochondria]. Postepy Biochem.

[45] Bitzur R (2016). Remembering statins: do statins have adverse cognitive effects?. Diabetes Care.

[46] Benarroch EE (2008). Brain cholesterol metabolism and neurologic disease. Neurology.

[47] März P, Otten U, Miserez AR (2007). Statins induce differentiation and cell death in neurons and astroglia. Glia.

[48] Guo Y, Zou G, Qi K, Jin J, Yao L, Pan Y, Xiong W (2021). Simvastatin impairs hippocampal synaptic plasticity and cognitive function in mice. Mol Brain.

[49] Cham S, Koslik HJ, Golomb BA (2016). Mood, personality, and behavior changes during treatment with statins: a case series. Drug Saf Case Rep.

[50] Golomb BA (1998). Cholesterol and violence: is there a connection?. Ann Intern Med.

